# Implementation of a novel secured authentication protocol for cyber security applications

**DOI:** 10.1038/s41598-024-76306-z

**Published:** 2024-10-28

**Authors:** V. Suresh Kumar, Osamah Ibrahim Khalaf, Radha Raman Chandan, Qusay Bsoul, Shashi Kant Gupta, Firas Zawaideh, Deema Mohammed Alsekait, Diaa Salama AbdElminaam

**Affiliations:** 1 Department of Computer Science and Engineering, Jaya Engineering College, Chennai, India; 2 Department of Solar, Al-Nahrain Research Center for Renewable Energy, Al-Nahrain University, Jadriya, Baghdad, Iraq; 3 Department of Computer Science, School of Management Sciences (SMS), Varanasi, 221011 UP India; 4 Cybersecurity Department, College of Computer Sciences and Informatics, Amman Arab University, Amman, 11953 Jordan; 5 Computer Science and Engineering, Eudoxia Research University, New Castle, USA; 6 Adjunct Research Faculty, Centre for Research Impact & Outcome, Chitkara University Institute of Engineering and Technology. Chitkara University, Rajpura, 140401 Punjab India; 7 Cybersecurity Department, Faculty of Science and Information Technology, Jadara University, Irbid, Jordan; 8 Department of Computer Science and Information Technology, Applied College, Princess Nourah Bint Abdulrahman University, P.O. Box 84428, Riyadh, 11671 Saudi Arabia; 9 MEU Research Unit, Middle East University, 11831 Amman, Jordan; 10 Jadara Research Center, Jadara University, 21110 Irbid, Jordan

**Keywords:** Authentication protocol, Attacks, Security, Spiking neural network with fuzzy logic (SNN-FL), Digital Age, Cybersecurity applications, Computer science, Information technology

## Abstract

Robust verification protocols are crucial for maintaining the security and reliability of sensitive information due to the increasing complexity of cyber-attacks. This paper introduces a novel 5G Secure Handover Protocol aimed at addressing security and effectiveness issues encountered in existing systems. The proposed protocol is robust against various attacks, including de-synchronization, replay, man-in-the-middle (MITM), denial of services (DoS), and jamming, ensures perfect forward key secrecy, safeguarding communication confidentiality. The proposed protocol utilizes a combination of spiking neural network and fuzzy logic (SNN-FL) techniques that must choose the goal cell as carefully as possible before initiating the transfer process. By combining fuzzy logic and spiking neural networks to reduce handover latency and thwart several types of cyberattacks, the proposed 5G Secure Handover Protocol improves security. Extensive simulations show its efficacy and emphasize its potential for safe communication in large-scale cybersecurity applications. The paper presents a novel secure authentication protocol that significantly reduces handover delays and improves efficiency. Simulations show its resilience against common security threats, protecting sensitive information and maintaining secure communication channels. The protocol, with low communication expenses, complex spatial, and latency for changeover verification, is ideal for large-scale cybersecurity applications, contributing to the development of secure digital authentication mechanisms.

## Introduction

The network improves how people access information, and technologies like the Internet of Things (IoT), multi-hop wireless networks, and wireless sensor networks (WSN) have significantly raised the amount of intelligence in people’s lives. Wireless and mobile communication have grown to be the most common forms of communication because of the advancement of telecommunications technologies like 4G, 5G, and Wi-Fi. In the meantime, people are increasingly managing their everyday activities through mobile terminals for various AI apps thanks to the trend of intelligence and the shrinking of equipment. The Global Mobile Network (GLOMONET), which provides mobile subscribers with roaming services, is a crucial infrastructure that allows subscribers to use the wireless network whenever and wherever they want^1^. The general structure of the GLOMONET environment is made up of three organizations: the Smartphone User (), the Overseas Agency () of the wandering network, and the domestic Agent (of the officially authorized network).There are substantial risks to the secrecy and safety of wireless network communications since the environment is so complex. As a result, authentication is a crucial GLOMONET technique. In the meanwhile, when the two parties have completed their bidirectional authentication, a session crucial understanding is also necessary for further safe communication. Additionally, the updating is of the time frame key a perfect feature for GLOMONET authentication if ongoing access is desired. The research separately identified the flaws in the approach, such as its inability to guarantee user anonymity and achieve backward secrecy. A malevolent insider might have used the anonymity element of the method in 2011, according to a deeper analysis of the flaws in the scheme^2^. Additionally, they thought the arrangement between and the session key was unjust and that it could be made by any party unilaterally. To achieve optimal security and equitable discussion-key consensusGLOMONET developed an authentication technique that preserves anonymity based on the Diffie-Hellman Protocol (DHP). Later, other researchers put out their own GLOMONET authentication techniques. The research in this area presented a strong verification protocol for GLOMONET, which utilized certificates to authenticate the three parties. Their plan had a single registration, no verification table, excellent efficiency, and resilience to attacks that would compromise smart card information. However, it was discovered that the protocol in could not reach a fair key agreement and was still open to a monitoring assault. It was suggested a DHP-based enhancement strategy to address these issues.Yet, compared to the prior system, theirs required simpler exponential processes^3^. Based on elliptic curve cryptography, an approachable GLOMONET security method. However, discovered that their plan had the same thing flaws as the plan presented a better GLOMONET security system in 2012. They were able to ensure forward secrecy and withstand man-in-the-middle attacks with their plan since it was built on the foundation of the inelastic curve DHP. However, their plan was vulnerable to several assaults and lacked some desirable features, such as local password changes. Several authentication mechanisms were subsequently suggested to boost security and efficiency. However, their protocol was unable to accomplish complete forward confidentiality since it lacked a session key update mechanism. Recently, Madhusudhan and Shashidhara drew attention to certain additional flaws in the method, including insider attacks, impersonator attacks, and attacks on seized verifiers. Additionally, they suggested a more reliable GLOMONET password system that they said was both safe and compact. However, their protocol’s design weakness meant that certain data that should have been kept private was instead sent in unencrypted. As a result, their system not only lacked adequate bidirectional authentication but also was vulnerable to user impersonator assaults, stolen validator assaults, handheld device breaches, and cyberattacks on session passwords. For GLOMONET, scientists have proposed several authentications for users’ techniques, however the bulk of these suffer privacy or usability concerns^4^. For instance, the majority of protocols failed to handle the conflict between the ability to change local passwords and resistance to attacks involving lost smart cards. Some verification data must be saved in the cellular phone or chip card to implement the local password-changing function. Therefore, while obtaining the smart card or smartphone, an opposition might utilize this verification information to deduce the related passcode. Cryptography using public keys is also necessary to guarantee the confidentiality of the key used for the session and secure updating of the session key. We provide a unique system of authentication with good security for GLOMONET to resolve this paradox and improve security^5^. To resolve the discrepancy between the alteration of the local passcode and the theft of Smart card fraud assault, the method of fuzzy verification approach is applied. To refresh the current time frame key, and maintain the session key’s security, a discrete logarithm-based DHP was used. Fuzzy logic and spiking neural networks were selected to improve GLOMONET security by bringing resilient and adaptable decision-making capabilities. By providing secure session key management and user authentication both essential for preserving confidentiality and thwarting illegal access in wireless network communications these technologies strengthen protocol resilience against a range of challenges.

## Contribution of this study.

- To innovative approach significantly reduces handover delays and improves the overall efficiency of the handover procedure.
- To evaluate the effectiveness of the protocol, extensive simulations were conducted.
- The results demonstrate its resilience against common security threats, highlighting its ability to protect sensitive information and maintain secure communication channels.

The study is organized as follows: Section II provides the literature review; Section III describes the proposed method; Sections IV and V discuss the outcomes; and Section VI concludes.

## Related work

Study^6^ examined present-day tactics. Thus, it categorizes solutions based on their methods of clustering, evaluates their application and limitations, discusses patterns, and identifies holes. According to the study, methods often seek one or more of the following four main goals: summary and sorting, pattern retrieval and processing, dynamic detection of outliers, and fluid identification of anomalies and sequences. Study^7^ looked at how cybersecurity applications take advantage of deep learning technology. DL, also called deep neural networks. Study^8^ discussed the issues surrounding utilizing deep learning techniques to aim for cyber security, including threat recognizing, modeling, monitoring, and research, as well as defense from numerous attacks safety measures, and delicate information. Study^9^ concentrated on recently developed DL methods for cyber security, including identification of intrusions, spyware, fraud and unwanted content, and vandalism of websites. An analysis of DL techniques for applications in cyber security is presented in this survey article. Deep automatic encoders, Restricted Boltzmann Machines, Recurrent Neural Networks, Generative Adversarial Networks, and several other DL techniques are all given a brief tutorial-style overview^10^. The offered [11] DL techniques for software related to cyber security including deep autoencoders, limited Boltzmann machines, recurrent neural networks, generative adversarial networks, and numerous more, is given a brief tutorial-style introduction. Research^11^ examined the safety of IoT in smart towns and discusses how these notions connect to knowledge and interpretation of Smart Cities (SC), Cyber Security (CS), and DL. Boltzmann machines, limited Boltzmann machines, deep belief networks, recurrent neural networks, convolutional neural networks, and generative adversarial networks were among the deep models for learning that we briefly covered. Paper^12^ addressed the issue of learning from unbalanced Networked information on the subject of safety online. A variety of balancing techniques are examined in addition to their results on various machine learning algorithms. A hypothetical human-in-the-loop intelligence cyber security model is offered based on the findings of this article, which also lists several limits and difficulties^13^. Study^10^ examined consideration it was invaded levels and security characteristics of cyber-security software; we explore variables impacting the desire to download safety apps. To do this, we offer an aesthetic that depicts the degrees of intrusion into privacy and the scope of various safety safeguards. Study^14^ examined typical cybersecurity vulnerabilities that will be found and analyzed. As a means of achieving this, a comprehensive map search was conducted, and a total of 78 main studies were found and examined. After carefully examining the selected study, we identified the key security flaws and their likelihood of occurrence. Data were also gathered and assessed to show the publication’s venue, nation of publication, and significant targeted infrastructures and applications. Study^15^ analyzed the author’s discussion of the use of machine learning techniques in the subject of cyber security while outlining the many model classifications based on their complexity, network-baseness, and learning process (supervised vs. unsupervised). Although authors consider all of the possibilities of artificial intelligence techniques, they focus on classification and forecasting while highlighting the possibility of improving algorithms to lower the rate of errors in created and distributed goods. Study^16^ highlighted the importance of several error criteria, such as the confusion matrix, mean absolute mistake, and relative error in regression and classification problems. In this review task, particular focus is also placed on the models’ applicability. There are several applications for these kinds of models, including recognizing intrusions and the detection and categorization of assaults, to mention a couple. Study^17^ offered a protocol for one-to-many identification that enables collaboration among senses for personal digital assistants (PDAs) as well as between each pair of sensor nodes when including or eliminating group members, the one-to-many verification technique is reliable and safe. It is important to note that the production of the group key and the procedure of member identification are simple. Research^18^ examined into account the ideal PUF environment; we first provide a lightweight privacy-preserving authentication mechanism for the RFID system. Subsequently, we present an improved procedure that can cope with the loud PUF environment. It is said that both of our protocols can get beyond the drawbacks of current models and additionally guarantee higher safety characteristics. Paper^19^ proposed Using larger chaotic maps and the “Fuzzy-Verifiers” and “Honey words” techniques, we propose a provably secure three-factor AKA method for portable, light gadgets. By maintaining the vulnerability of the generalized chaotic-maps Computation Diffie-Hellman issue, we demonstrate the integrity of the suggested protocol UN the arbitrary oracle context. Study^20^ demonstrated that this combination can assist in eliminating this necessity; they construct verification and key exchange protocol by integrating the concepts of Identity Encryption (IBE), PUFs, and Key-ed Hash Function. The Session Key Security and the Universal Composability Framework provide formal proof of the protocol’s integrity. Study^21^ provided a thorough examination of the available edge-based IoT security choices; the study aims to serve as a source of ideas for brand-new edge-based IoT security solutions. They first propose an edge-centric IoT architecture.Then, they carefully examine the edge-based IoT safety research activities in the context of safe designs for IDS, authentication and authorization procedures, and confidentiality strategies. The study occurred if we permit users to get real-time data from moving drones within the IoD environment without going via a server. This is a major security issue that might harm the effectiveness of any solutions used in this IoD environment. Study^22^ proposed taxonomy and categorization based on the application domain and underlying system architecture and provided an overview of IoT security threats. We also discuss several key IoT characteristics that render it difficult to develop robust safety mechanisms for apps that use the Internet of Things.

## Methods

### System representation of our protocols

#### *Handover triggering parameters*

This work proposes a multi-criteria changeover and key handling technique, where the transfer of keys triggered factors includes acquired carrier capacity, power volume, route loss, communication intensity, call blockage likelihood, and acceleration. The chosen model for acquired power $\:{B}_{k\:}$is given in (1):

1 $$\:{B}_{k\:}=20\:log\left[\frac{\lambda\:}{4\pi\:t}\right]+{B}_{d}+{S}_{d}+{S}_{k\:}$$

where$\:\lambda\:$represents the signal’s frequency in meters., $\:t$ is the gNBUEmeasured in meters, $\:{B}_{d}$ is the transmission level in dBm,$\:{S}_{d}$the gain of the gNB antennas $\:{S}_{k\:}$UE antenna performance as opposed to that, (2) demonstrates the force of intensity$\:{B}_{T}$model:

2 $$\:{B}_{T}=\frac{{B}_{d}{S}_{d}}{4\pi\:{K}^{2}}u/{n}^{2}$$

Here, $\:K$is is the distance that extends from the bottom of the subscription to the topmost point of the gNB. The customized Stanford University Interim (MSUI) approach, that is stated as given in, was used to calculate route loss (3):

3 $$\:{B}_{F\left(NGWJ\right)}=\alpha\:\left({B}_{F\left(GWI\right)}\left(t\right)-{B}_{F\left(GWI\right)}\left({t}_{0}\right)\right)+{B}_{F}\left({t}_{0}\right)+G$$

where $\:\alpha\:$the incline adjustment coefficient (the same as 0.88), $\:G$is the shadow compensation in dB $\:8.2<G<10.6\:tP,{t}_{0}$is a standard length (in this particular case, one meter), $\:{B}_{F}\left({t}_{0}\right)$is the distance field’s loss of pathway that gNB antenna,$\:{B}_{F\left(GWI\right)}\left(t\right)$SUI lost path concept is described in (4):In (4), $\:E$xisting space-free route degradation in $\:tP,\:x$is the magnitude of the route cost,$\:{Y}_{l\:}$the adjustment for wavelength 940 in MHz, and $\:{Y}_{z}$is the meters-based adjustment ratio for the reception antennae length. Regarding traffic intensity $\:{D}_{j\:}$(4) provides the accepted model:

4 $$\:{D}_{j\:}=\gamma\:\mu\:$$

The one in question was Erlang’s blocked hazard estimate C formula given by (5):

5 $$\:{B}_{p}=\frac{\frac{{E}^{M}M}{M!M-E}}{{\sum\:}_{j=0}^{M-1}\frac{{E}^{j}}{j!}\frac{{E}^{M}M}{M!M-E}}$$

Here$\:M$ and $\:E\:$are the quantity of provided traffic and the total amount of pathways, accordingly.

### The integration of the proposed protocol using spiking neural network and fuzzy logic techniques (SNN-FL)

In the context of integrating spiking neural networks (SNNs) with fuzzy logic (FL) approaches, SNNs are employed for pattern recognition and temporal spike train-based decision-making, while fuzzy logic evaluates uncertainties in security assessments. SNNs are used in this protocol to evaluate inputs and produce outputs based on neural activity, and fuzzy logic is used to describe the security requirements using membership functions and fuzzy rules. This allows for a more thorough evaluation of component security inside the 5G architecture.

#### *Spiking neural networks*

The development of SNNs as a potent third-generation neural network in recent decades has sparked several studies with an emphasis on biologically inspired methods for identifying patterns. SNNs were first influenced by the neurological system and the way neurons communicate with one another to change input using discrete action potentials in time using adjustable connections. Whenever the cumulative total of the prospective shift of the membrane, which can be caused by postsynaptic entertainment, passes a threshold in the human neuron, a voltage spike is produced. The frequency of peak emergence and the chronology of spike trains reveal both current calculations and external cues. The methods for creating spikes and transforming information are fairly comparable in SNNs. The specifics of SNN designs and the learning techniques used with these kinds of nets are covered in 4 Algorithm 1.

**Algorithm 1:**
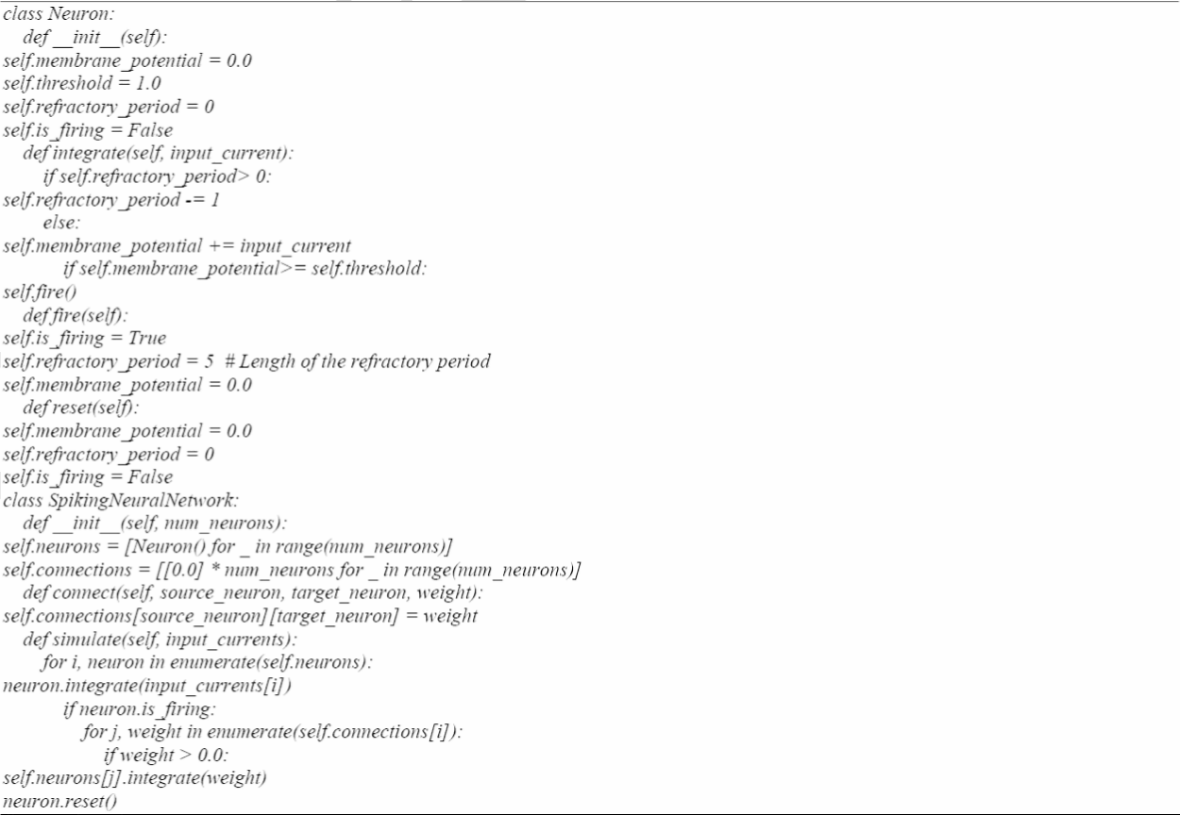
SNN Algorithm

##### SNN architecture

Spiking neurons and linking synapses that may be described by varying scalar weights make up an SNN structure. Encoding analog input data toward the spiked train via a rate-based approach, a kind of spatial encoding, or community coding is the first stage in putting an SNN into practice. A physiological in cerebral neurons gets inputs called synaptic from the other neurons in the neural network, as was previously mentioned. System dynamics and action potential generating dynamics both exist in biological brain networks. Compared to genuine biological systems, synthetic SNNs’ connectivity patterns are significantly more straightforward. Assuming the simulations of firing neurons show simple threshold characteristics in this situation is helpful. A potential of action or spike is produced when the neuron with a postsynaptic potential in the membrane passes a threshold as a result of the function of postsynaptic synapses, which alters the membrane potential. Hodgkin and Moore were the pioneers in these occurrences modeling. Due to pre-synaptic neuron activity, which affects the cells up-to-date a potential for action or spike is created when the potential at the membrane of post-synaptic neurons crosses a threshold. Hutchinson and Moore were the initial ones to simulate these events. The LIF model is very well-liked since it accurately depicts the intuitive characteristics of outside input collecting over a barrier that is leaky in cells with a distinct threshold. Synaptic connections allow the propagation of pulse sequences in firing neuronal networks. The synapse can be either excitatory or inhibitory, affecting the potential at the membrane of the neurons differently depending on the data that it receives. As a result of learning, the power of the adaptable synaptic (weights) might alter. Because the non-differentiability of spike sequences restricts the commonly employed backward propagation approach, the initialization algorithm of an SNN is a particularly difficult part to build for multi-layer (deep) SNNs.

##### Learning rules in SNNs

As was already established, learning is accomplished in almost all ANNs, spiking or nonspiking, by modifying the weights of synaptic neurons with values for scalars. Spiking allows for a specific bio-plausible training rule that can’t be easily reproduced in networks without spikes. The broad term “spike-timing-dependent polymorphism” (STDP) refers to several variations of this learning principle. Its distinguishing characteristic is that the weight between pre- and post-synaptic neurons is altered by their respective spike timings within a temporal window of about tens of milliseconds. The data utilized to modify the weight is both temporally and locally specific to the synapse. The common unsupervised and supervised methods of learning in SNNs are described in the next segments.

### Unsupervised learning via STDP

As was previously mentioned, STDP frequently functions as a learning process in autonomous training in SNNs. The most typical explanation for biological STDP is fairly simple. The weight holding the two neurons together is made stronger if ahead of the presynaptic cells, a posterior neuron briefly becomes active. The logic is weaker and the presumption of causation between the two processes if a presynaptic neuron activates, something is awry after the postsynaptic neuron has finished firing. A long-term pot (LTP) is the word for strengthening; declining is referred to as long-term depression (LTD).

The expression “long-term” is employed to differentiate among extremely brief impacts on the rating system of a few ms that are demonstrated in testing. For a single spike set created by matching to real data, Formula (6) idealizes the most often observed STDP rule in experiments.

6 $$\:=\left\{\begin{array}{c}{Ea}^{\frac{-\left(\left|{d}_{pre}-{d}_{post}\right|\right)}{\tau\:}{d}_{pre}-{d}_{post}}\le\:0E>0\\\:{Aa}^{\frac{-\left(\left|{d}_{pre}-{d}_{post}\right|\right)}{\tau\:}{d}_{pre}-{d}_{post}}>0E<0\end{array}\right.$$

The framework was created with a modicum of biological validity in mind.Their network’s STDP rule is depicted in (Eq. 6). If the presynaptic neurons fire briefly (for example, within = 10 ms) preceding the postnatal neuron, LTP will take place. If not, LTD happens.The E-step of the EM method, which produces an example of the encoded concealed anterior probability variables, is caused by a result neuron firing a spike. The M-step in EM is defined by the delivery of triggered output neurons’ synapses via STDP. To execute the WTA using an inhibitory neuron, this increased the model’s suitability for implantation in a cerebral microcircuit.

7 $$\:\varDelta\:{u}_{rj}=\left\{\begin{array}{c}{a}^{-{u}_{rj}}-\text{1,0}<{d}_{r}^{l}-{d}_{j}^{l}<\epsilon\\\:-1,\:\:\:\:\:\:\:\:\:\:\:\:otherwise\end{array}\right.$$

There has been a lot of debate regarding whether backpropagation training can be done in the brain directly from a biological perspective. There are two significant problems with SNNs, as may be seen from the calculation below. The chain rule yielded the following fundamental formula, which appears in all backpropagation models:

8 $$\:{\delta\:}_{i}^{\mu\:}=s{\prime\:}\left({e}_{i}^{\mu\:}\right)\sum\:_{r}{u}_{rj}{\delta\:}_{i}^{\mu\:}$$

In the previous illustration, just a piece of the pattern of inputs for the cost curve about the net output to any arbitrarily chosen unit is represented by $\:j$ and $\:k$. The group of elements determined by $\:k$will get direct feedforward connections from unit $\:j$. The unit’s net input, designated as aj, is subjected to activation using the function. From unit $\:j$, the feedback loadsto the group of units that $\:k$ indexes are represented by$\:{x}^{d}Y$.The Widrow-Hoff (Delta) rule, which was first applied to non-spiking linear units, is modified for SNNs by ReSuMe. The planned production minus the actual output determines how much the Widrow-Hoff rule weights vary, as indicated below.

9 $$\:\varDelta\:u=\left({x}^{d}-{x}^{o}\right)Y={x}^{d}Y-{x}^{o}Y$$

Where $\:{x}^{d}$ and $\:{x}^{o}$ are the intended and observed outputs, and $\:x$ is the presynaptic input, respectively. The combined STDP and anti-STDP can be used to express the rule when enlarged as depicted on the RHS and reformed for SNNs. In other words, the instruction for excitatory synapses has the following form

10 $$\:\varDelta\:u=\varDelta\:{u}^{STDP}\left({G}^{in},{G}^{t}\right)+\varDelta\:{u}^{eSTDP}\left({G}^{in},{G}^{0}\right)$$

In the example above, STDP depends on the relationship between the intended and presynaptic when spike railways depending on desired and observed spike trains. There isn’t a direct physical link since the educational rule relies on the relationship between the instructor neuron and the input neuron. This is the reason the phrase “remote supervised learning” contains the word “remote.” Even though the learning must align with regular STDP qualifying periods, the fact is that it is not clear from the equation above.

The Widrow-Hoff rule serves as the foundation for the SPAN model’s learning algorithm. Rather than amending the legislation to create an SNN, SPAN uses a digital-to-analog transformation to make this SNN compatible with Window-Hoffspikes sequences utilizing alpha kernels of the type $\:t$. All spikes are converted to at this point, a linear accumulation of PSPs since this is a typical formula for simulating posting synaptic potential. This is comparable to the method used in SpikeProp, which was mentioned starting with the present section. Then, the idea of learning can be stated as

11 $$\:\varDelta\:u\propto\:\int\:{\stackrel{\sim}{Y}}_{j}\left({\stackrel{\sim}{X}}_{t}\left(d\right)-{\stackrel{\sim}{x}}_{o}\left(d\right)\right)td$$

Where the constraints for inclusion involve the pertinent timezone time and a tilde identifies the analogous structure of the spiking spikes.

### Fuzzy logic for evaluating security of component

Fuzzy logic is employed in the suggested study effort to assess the part security. In unclear situations, this strategy is really helpful. It may be used for several things. The method of assessing an element’s security is called part safety assessment. A method for evaluating components of high caliber satisfies the security requirements. The proposed technique is described in more detail below.

#### *Fuzzy logic*

Different issues have been resolved using fuzzy set theory in a variety of domains. Since it addresses the issues of ambiguity and imprecision, engineering is where it is most frequently utilized. This tool aids in giving solutions for issues that are challenging to analyze. It comprises several inputs and a Membership Function (MF), and a model of fuzzy rules is created used to model and assess the element’s security. The various MFs that are used as inputs include: control of access (no access control, substance accessibility management, and full accessibility control), authorization (low verification, there are two levels of authentication: high and medium), non-repudiation (no rejection, small rejection, and excessive non-repudiation), confidentiality of data (no confidentiality of data, substance data anonymity, and significant data anonymity), and flow of communication (no access control, small access management, and packed access control).

#### Handover and Key Management

The functions of authentication computer, authentication credential repository and processing, and access and mobility management (AMF, ARPF, and AUSF) were every participant in the inside new radio handover (intra-3GPP) simulation over actual transfer and handling of keys. The 3GPP-defined traditional 5G architecture is preserved by this protocol. In this instance, the KAMF key at the AMF and UE determines the next hop parameter or NH. K stood for the permanent secret code that was previously stored in the universal subscriber and the ARPF, and the AMF provided key management in addition to authentication with the UE serving as the AUSF and ARPF. An identity module is the USIM. The target AMF (which used K’AMF to interact with UE) was marked as TAMF, while the source of AMF was given the SAMF designation.

## Results and evaluation

The efficacy of the designed protocols is about communications costs; this part includes the modeled parameters as well as information on geographic complexities, fallback latencies, and the number of executed transactions.

### Simulation parameters

Table 1 displays the simulation settings used in this study. A mixture of the RD (random direction) and RWP (random waypoint) models was employed to create the mobility model.

**Table 1:** Parameters for simulation.

Parameter	Value	Units
changed referencing range SUI, $\:{t}_{o}$	2	meters
Slope correction factor, $\:\alpha\:$	0.89	-
Shadowing correction, $\:G$	9.3	$\:tP$
Reference distance for SUI, $\:{t}_{o}$	2	Meters
Maximum eNB-UE distance, $\:t$	249	meters
Transmission Frequency, $\:l$	29	Szh
Transmitter antenna height, $\:z\:or\:{z}_{d}$	52.6	Meters
gNB Transmit power, $\:{V}_{d}$	21	$\:tPn$
Subscriber height, $\:{z}_{0}$	1.6	Meters
Mobility model	RD & RWP	-
Correction for frequency, $\:{Y}_{l}$	-11.6	MHz
Transmitter antenna gain$\:{S}_{d}$	19.3	$\:tPj$
Free space path loss, $\:E$	41.39	$\:tP$
Path loss exponent, $\:x$	3	-
Correction for receiving antenna height, $\:{Y}_{z}$	34.2	Meters

### Communication overheads

**Fig. 1:**
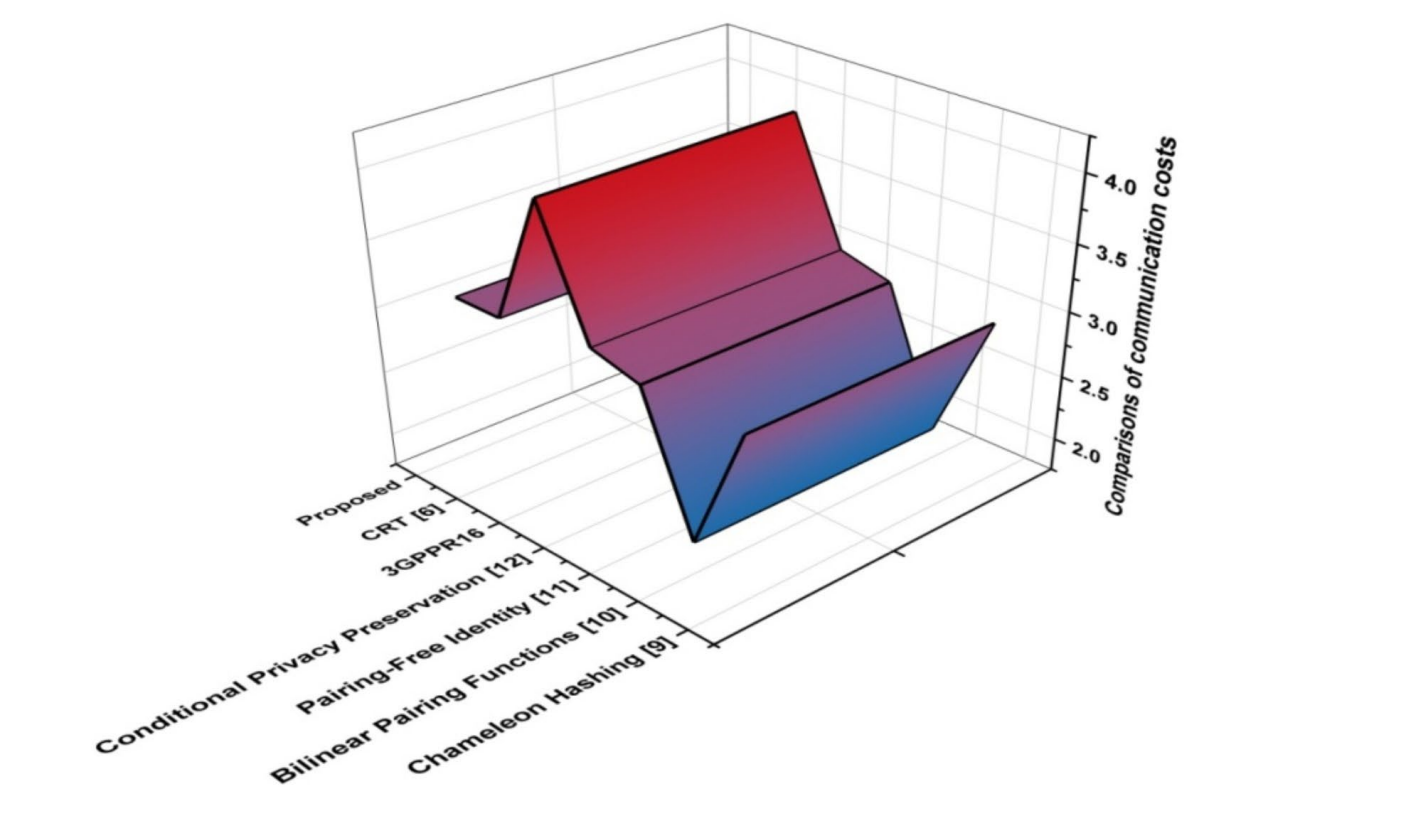
Communication costs Comparisons.

The suggested protocol’s communications expenditures are displayed in (Fig. 1). Then, they are compared to the identical 3GPP R16 specifications and the recommended methods. Here, it is demonstrated what the communication costs for the 3GPP R16 and suggested protocols were. These findings showed that the overheads of the suggested protocol were comparable to those of the schemes.Table 2 depicts the Analysis of Communication cost Comparisons.

**Table 2:** Analysis of communication costs comparisons.

Comparisons of communication costs	
Chameleon Hashing [9]	3
Bilinear Pairing Functions [11]	2
Pairing-Free Identity [11]	3
Conditional Privacy Preservation [12]	3.1
3GPPR16	4
CRT [6]	3
Proposed	3

The 3GPP R16 had the largest overhead for communicating, which was 4, while a protocol based on bilinear pairing algorithms has the lowest communications cost.The UE, $\:{S}_{g}$NB, and TgNBdivided up the three costs for the entire scheme. The UE, $\:{S}_{g}$NB, and TgNB shared the first two overheads and the final overhead in the case of the proposed technique and the one now in use. As a result, compared to 3GPP R16, the suggested using the methodology, a 25% reduction of transmission overheads.

### Space complexities

**Fig. 2:**
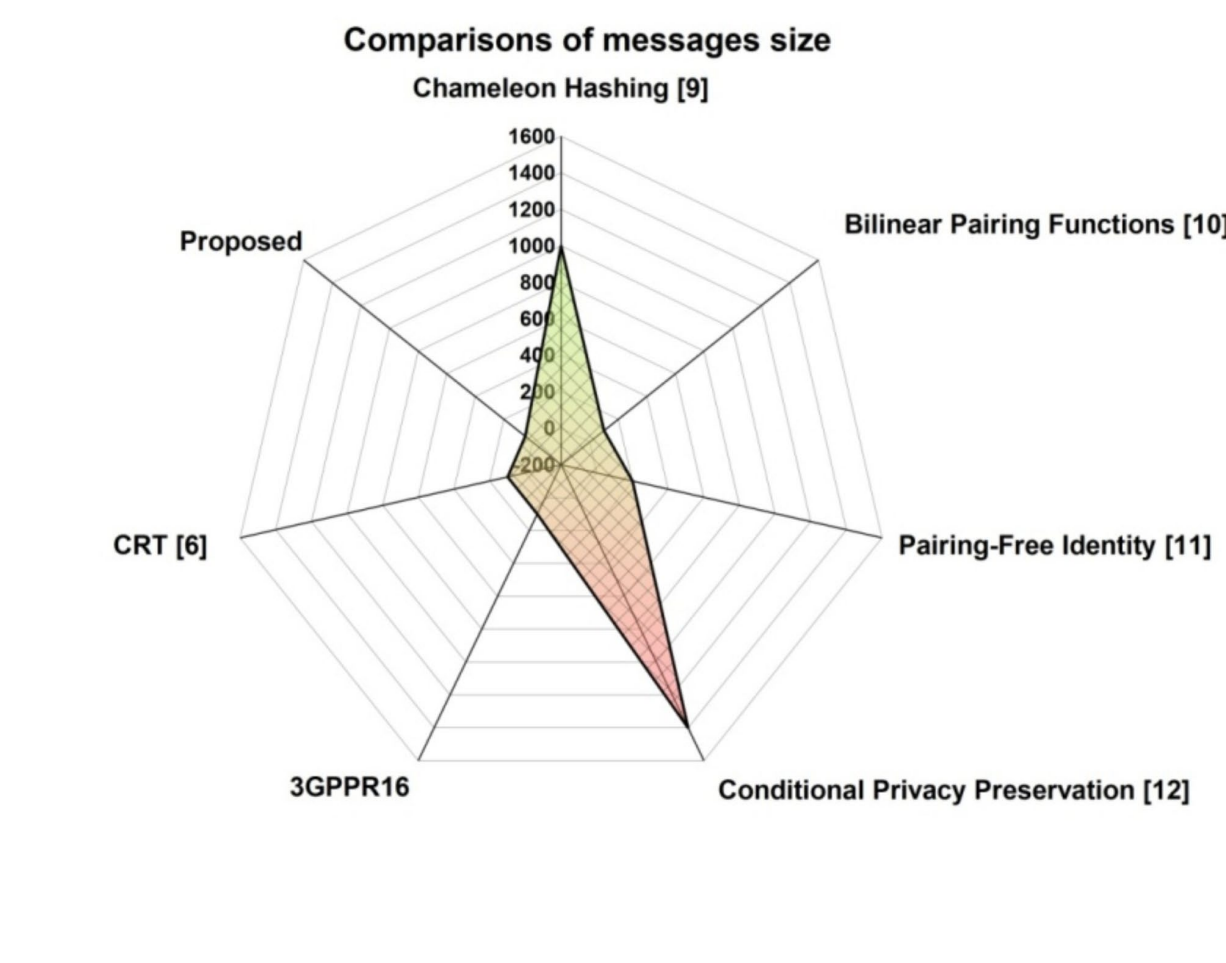
Message Size Comparisons.

In terms of space-intensive contrasts, the overall message sizes for the 3GPP R16 both the recommended protocol and methods 108, 1072, 80, 160, 1348, 112, and 64, respectively. In (Fig. 2), contrasts between these outcomes are displayed. Table 3 shows the Analysis of Messages Size Comparisons.

**Table 3:** Analysis of message size comparisons.

Comparisons of message size	
Chameleon Hashing [9]	1000
Bilinear Pairing Functions [11]	100
Pairing-Free Identity [11]	200
Conditional Privacy Preservation [12]	1400
3GPPR16	100
CRT [6]	100
Proposed	50

The suggested protocol, as demonstrated here, has the smallest total message size. The suggested protocol lowered the overall difficulty by 42.9% when compared with 3GPP R16. 5G connections require extremely effective and straightforward handover authentication techniques because they happen to be both resource- and power-constrained. As a result, our protocol met these criteria thanks to its efficiency in terms of the amount of memory needed to execute the entire handover.

### Handover latencies

**Fig. 3:**
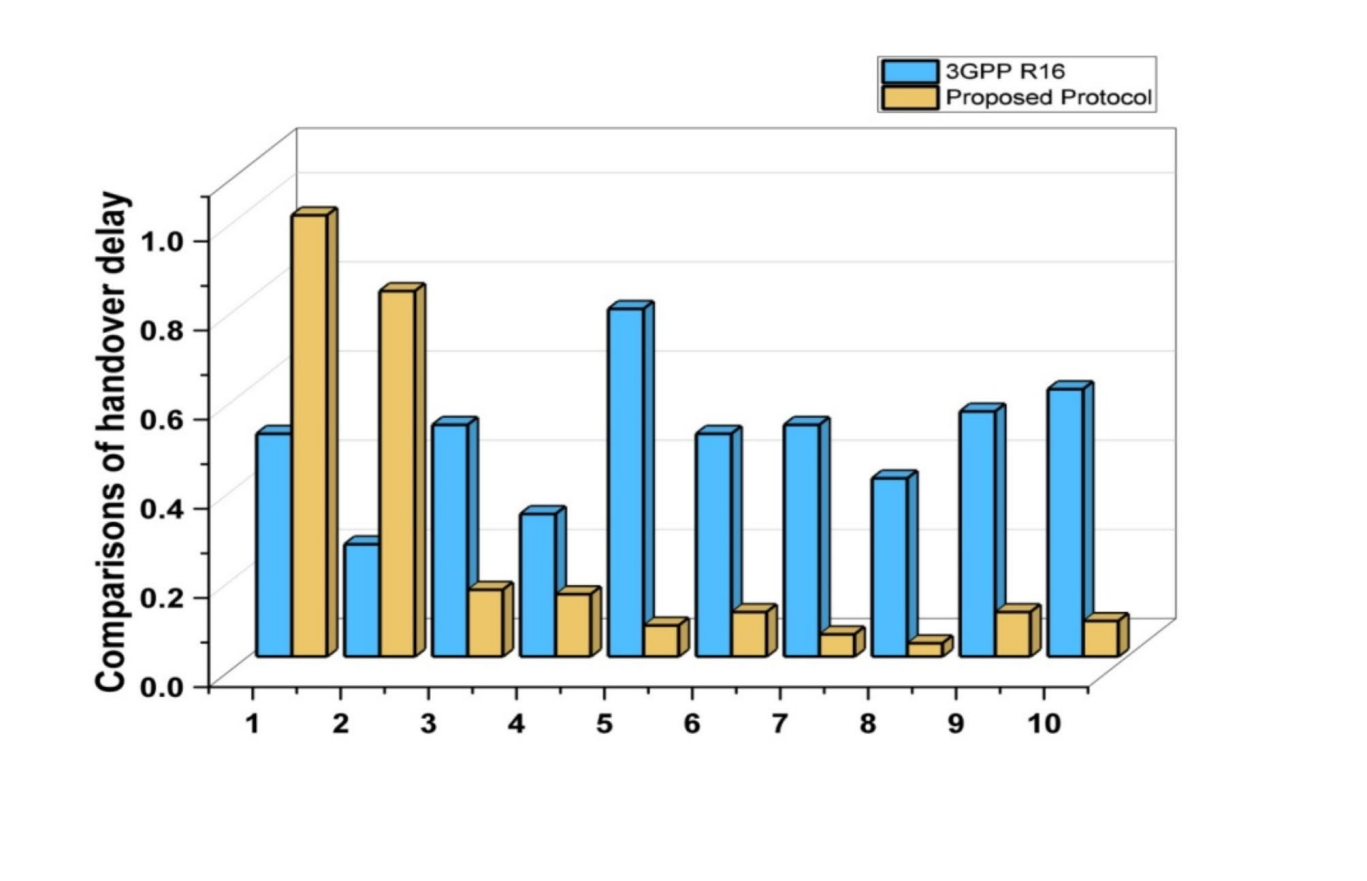
Handover Delay Comparisons.

The efficiency of the protocol that was suggested was contrasted to that of 3GPP R16 based on changeover latencies, and the outcomes depicted in (Fig. 3). The suggested protocol has shorter handover latency than the 3GPP R16 for all simulated rounds. Table 4 shows the Analysis of Handover Delay Comparisons.

**Table 4:** Analysis of Handover Delay comparisons.

Comparisons of handover delay		
	3GPP R16	Proposed Protocol
1	0.5	0.99
2	0.252	0.82
3	0.52	0.15
4	0.32	0.14
5	0.78	0.07
6	0.5	0.1
7	0.52	0.05
8	0.4	0.03
9	0.55	0.1
10	0.6	0.08

Earlier network variable measurements and the use of the SNN-FL approach in choice contributed to the decrease in changeover delays. In the end, this is consistent with the 5G wireless network standards’ goals.

### Number of executed handovers

**Fig. 4:**
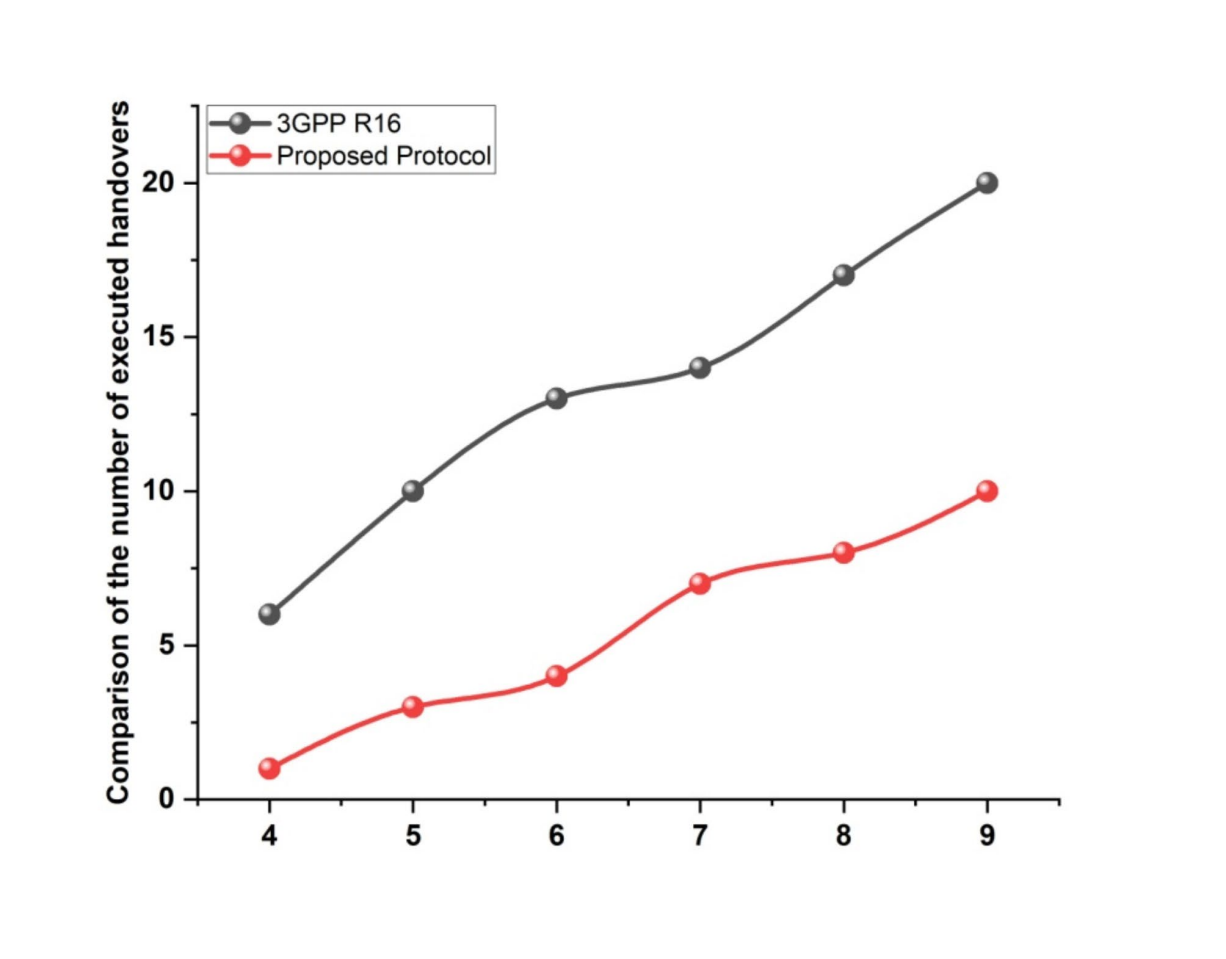
Comparison of the number of executed handovers.

In terms of the quantity of completed handovers, (Fig. 4) compares the suggested procedure. As seen in (Fig. 4), the suggested protocol performed fewer handovers than the standard 3GPP R16.Table 5 shows the Analysis of Comparison of the number of executed handovers.

**Table 5:** Analysis of comparison of the quantity of handovers that were completed.

Comparing how many handovers were really completed		
	3GPP R16	Proposed Protocol
4	6	1
5	10	3
6	13	4
7	14	7
8	17	8
9	20	10

As a result, the number of transitions was maintained to a minimum, which reduced the risks associated with the process of handing over. This was accomplished by integrating several criteria as inputs to the turnover-triggering decisions.

### Assessment of security and privacy

The recommended processand its renewal after each transfer aids in preventing monitoring attacks. In contrast, GUTI has not been modified for a long time in the present 5G environment. The likelihood of de-synchronization attacks was reduced since the management of keys was carried out using an inclined key generation method rather than a horizontal methodology. NCC was contained in EE2, EE3, EE4, and EE5, secured via SK, and its recentness was confirmed by both random values and agreed timestamps. Additionally, safeguarded its communication from AMF to UE. Reverse key confidentiality is assured because in the present As a result, TgNB can never compute the Key for sessions utilized by $\:{S}_{g}$NB. However, because TgNB uses KgNB* directly as its KgNB, until it is refreshed, $\:{S}_{g}$NB is aware of this session key (forward key secrecy is not ensured)sets NCC to an extremely high value (and transmits it to T$\:gNB$ as in step 21), desynchronizing T$\:gNB\:$and preventing crucial agreement. If necessary, the UE continues step 29, increasing the local NCC by 1 till it corresponds to the recognized NCC. As a result, new key generation requires more time. The handover fails because this derived KgNB* is distinct from the $\:{k}_{g}$NB* used by $\:{T}_{g}$NB since it utilizes a different NH (incremented NH). A DoS occurs as a result of the UE being unable to generate $\:{k}_{g}$NB * to finish the transfer of control. Replayed assaults are also possible since an attacker pretending to be S$\:gNB$ possesses $\:{k}_{g}$NB * and NCC obtained in earlier handovers that may be repeated for the next changeover. As it lacks KAMF and $\:{k}_{g}$NB in our protocol, $\:{T}_{g}$NB is unable to deduce the first NH (step-12). Additionally, $\:{T}_{g}$NB lacks knowledge of $\:{k}_{g}$NB* and is unable to deduce it (because only AMF is capable of doing this), assuring backward key secrecy (since $\:{T}_{g}$NB is unable to infer past handover keys). Additionally, GUTI is refreshed during the handover procedure, which causes the received KgNB* to be updated in line with step-33. As a result, step 20 cannot be used by $\:{S}_{g}$NB to determine $\:{T}_{g}$NB$\:{k}_{g}$NB. This successfully prevents replay attacks, together with timestamps. Additionally, contains time stamps in other parameters, the next step is encryption, making it impossible for an attacker to intercept and change them. MITM attacks are thwarted by mutual recognition amongst the handover entities (UE, $\:{S}_{g}$NB, and $\:{T}_{g}$NB) before the creation of an account key. Additionally, because NH and KAMF keying materials are computed individually at AMF and never transferred via a network, an opponent cannot listen in on them.

### Evaluation metrics

The evaluation of the proposed SNN-FL technique in terms of Accuracy, Precision, Recall, and F1-Score gives better outcomes when compared to other existing methods. Comparative analysis with other existing methods is Improved K-nearest neighbor (Improved K-NN) ^[[23]]^, Fuzzy-Q Learning ^[[23]]^, Support Vector machine-K-nearest neighbor-Linear regression (SVM-KNN-LR)^23^. Table 6 shows that the performance of various parameters.

**Table 6:** Outcome of metrics.

Methods	Accuracy (%)	Precision	Recall	F1 Score
Improved KNN ^[24]^	92	0.91	0.92	0.90
Fuzzy-Q learning ^[24]^	89	0.82	0.83	0.85
SVM-KNN-LR ^[24]^	96	0.96	0.96	0.96
SNN-FL [Proposed]	98	0.97	0.97	0.98

**Fig. 5:**
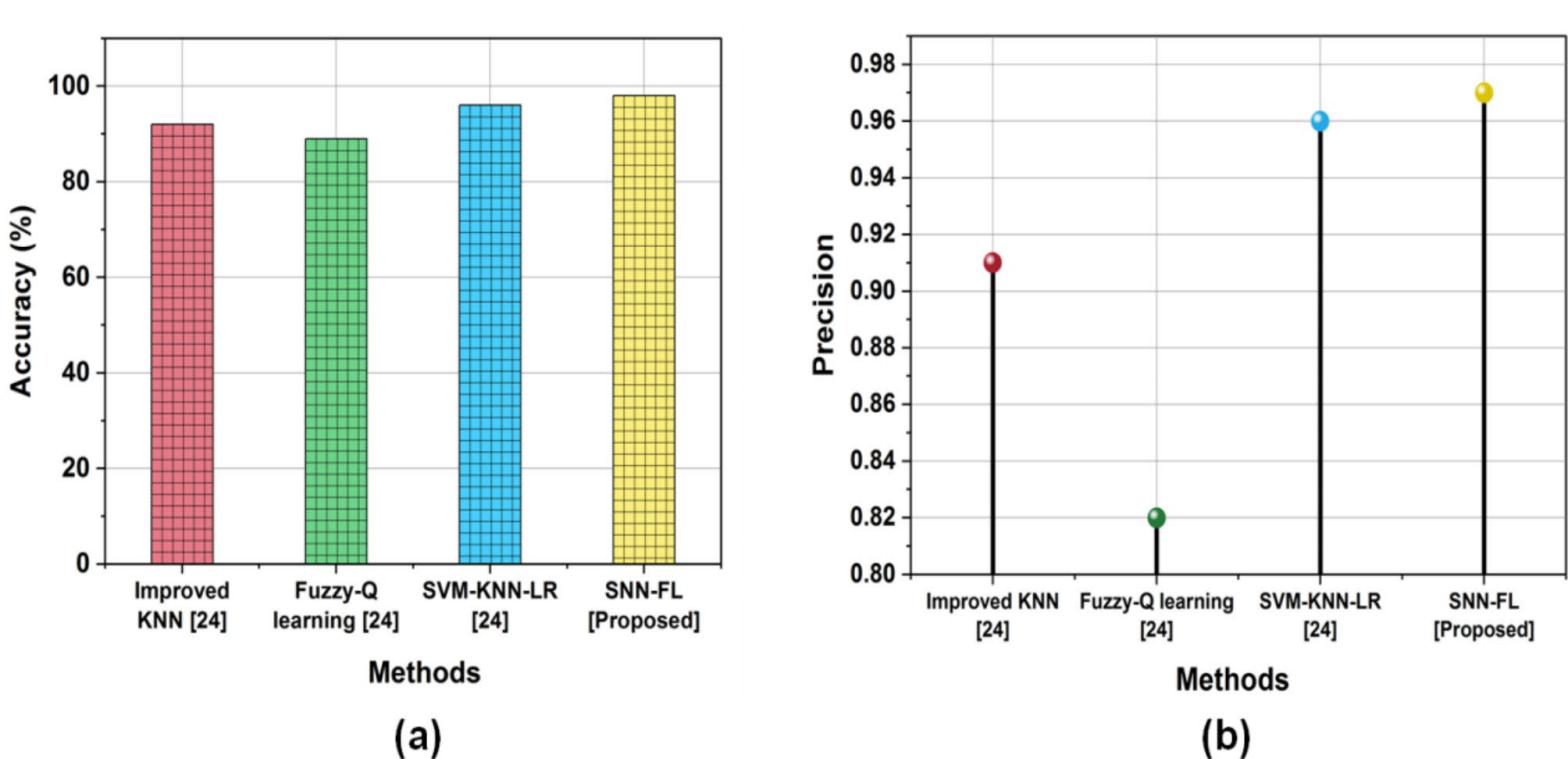
Comparison of (**a**) Accuracy, (**b**) Precision.

The accuracy metric calculates how accurate the model’s predictions are overall. It is determined by dividing the total number of samples in the test set by the number of samples that were properly categorized. A key metric used to assess performance in the domains of statistics and ML is precision. Out of all samples labeled as positive, precision represents the percentage of accurately diagnosed positive samples. (Fig. 5) shows the comparison of Accuracy and precision with proposed and existing techniques.

In comparison, the proposed SNN-FL method achieved better accuracy (98%) when compared with other existing methods of Improved KNN (92%), Fuzzy-Q learning (89%), SVM-KNN-LR (96%), and precision providing better outcomes of the proposed method of SNN-FL (0.97).

**Fig. 6:**
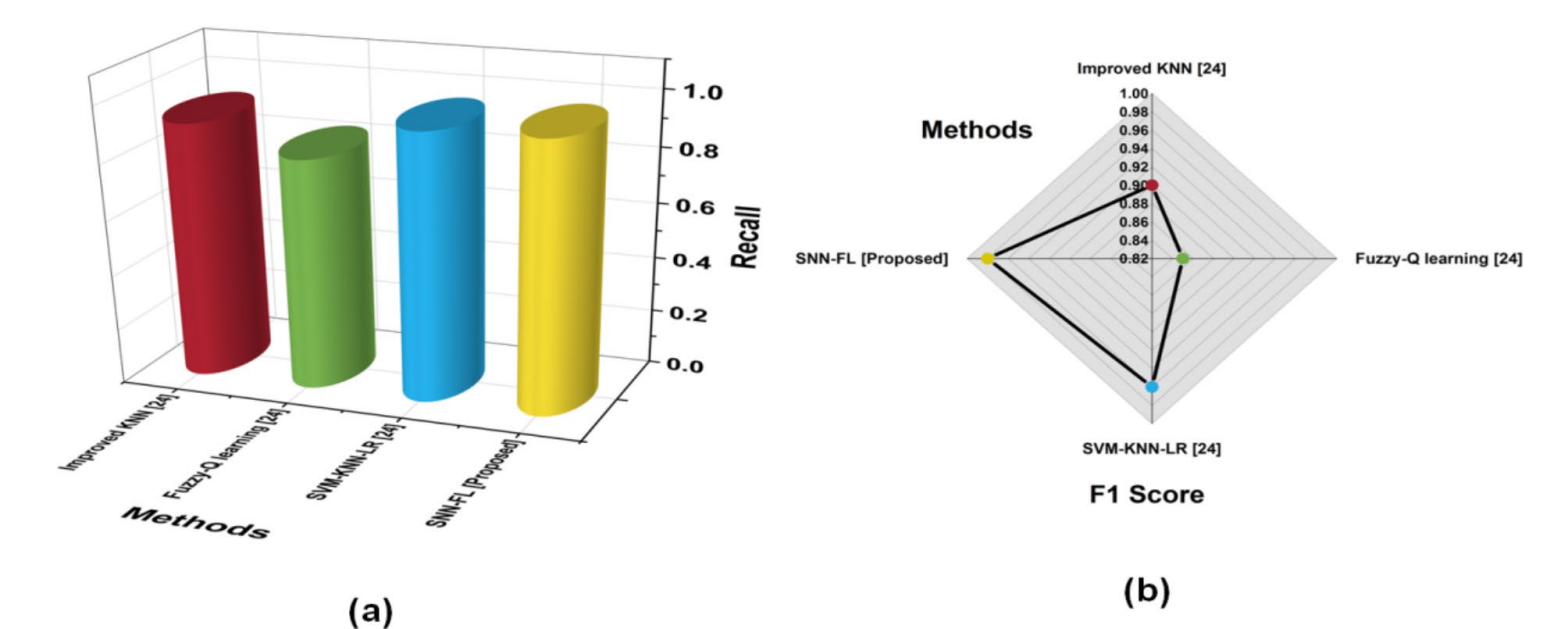
Comparison of Recall and F1-score.

Recall is a performance statistic that shows the percentage of real positive sentiments that a model correctly detects and is used in data classification and machine learning. The capacity of a model to discern between positive and negative attitudes is enhanced by the F1-score. Sentiment classification skills are evaluated on both false positives and negatives. (Fig. 6) shows the comparison of Recall and f1-score with proposed and existing techniques.

When compared to other current approaches, the suggested SNN-FL method produced higher results in terms of Recall (0.97) and precision (0.98).

Testing the suggested SNN-FL approach in a variety of cybersecurity circumstances, including intrusion detection, malware classification, and threat investigation, can show how adaptable and cooperative it is. The SNN-FL approach that has been suggested exhibits flexibility and scalability to many cybersecurity applications because of its strong examination metrics, which include accuracy, precision, recall and F1-score. Its better performance over previous technologies ensures compatibility with existing systems and suggests possible interoperability and efficacy in many cybersecurity situations.

### Average packet drop rate

**Fig. 7:**
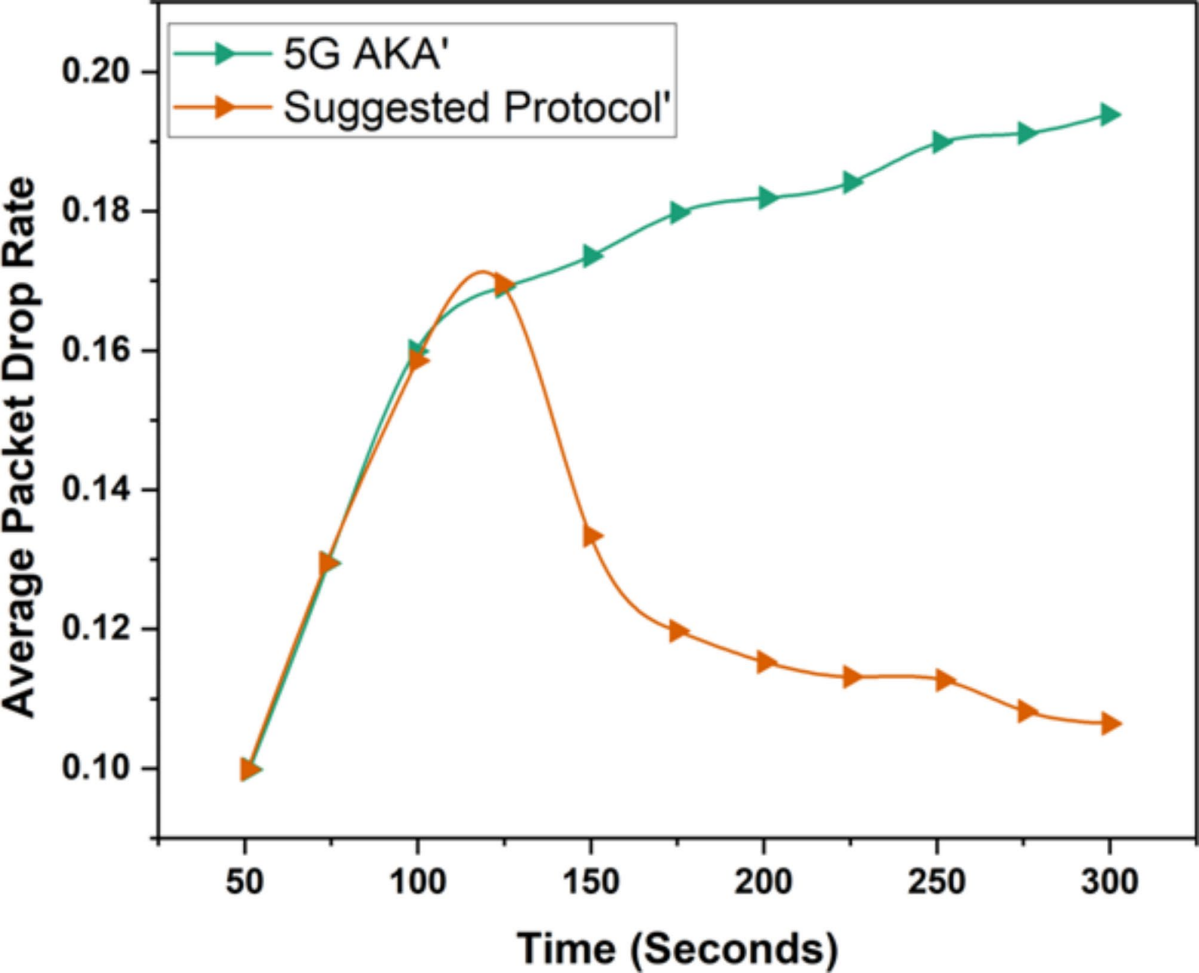
Average packet drop rate.

In 5G AKA secure handover protocols, the standard packet drop rate is a vital performance metric that indicates how well associations are transitioned linking cells. Attacks from cyber security, like MIMD and DOS, can cause a hugequantity of packet drop rates by intrusivestatements, which can result in data loss and poor network performance. Therefore, it is vital to have strong security events in place to diminish these risks and maintain optimal packet communication during handovers.(Fig. 7) shows the 5G AKA and our suggested protocol average packet drop rate outcomes are shown.

## Discussion

The recommended protocol displays competence in conditions of statement costs, space difficulty, and handover latencies while exhibiting flexibility against a range of attacks, such as de-synchronization, MITM, replay, DoS, and jammer attacks. A systematicassessment of its potentialdrawbacks and weaknesses is necessary for decisiveexpediency. One important limitation is the protocol’s reliance on exact mobility models, such as RD and RWP, which candelay its applicability in real-world contexts with variedassociationpatterns. In addition, the key allocation and managementevents might contain unrevealed vulnerabilities, potentially revealing the procedure for a novel attack strategy. The difficulty and dispensationload of the protocol also pose a disadvantage, creation it inappropriate for plans with limited belongings. The assessment graph of the existingapproach, including Improved K-NN, Fuzzy-Q Learning, and SVM-KNN-LR ^[[23]]^, highlights these limitations. Improved K-NN, despite its efficiency, frequently incurs high processing costs and is vulnerable to noise. Fuzzy-Q Learning, while flexible, tends to be also highly exact or recall-oriented, potentially most important to misclassifications. SVM-KNN-LR, though extremely accurate, is computationally difficult and complex. In contrast, the proposed SNN-FL approach considerably enhances these metrics, achieving a precision of 0.97 and an accuracy of 98%. By effectively lowering computing complexity, boosting noise resilience, and guaranteeing more precise and trustworthy classification, this advancement is made possible. Despite these benefits, a thorough assessment of the protocol’s versatility and possible weaknesses is required to properly determine its effectiveness and resilience in a range of real-world applications.

## Conclusion

It has been demonstrated that 5G-AKA’s several flaws make it vulnerable to a variety of attacks, including MITM, DoS attacks missing key verification attacks, tracing attacks, replay attacks, desynchronization attacks, and jamming attacks (using modified NCC). It has been shown that the new protocol can thwart some of these assaults, and because it maintains the standard 5G architecture and its implementation is simple. This protocol performs well because of its little space complexity and handover latency. The findings show that our proposed SNN-FL techniques achieved better outcomes of accuracy of (98%), precision of (0.97), recall of (0.97), and f1-score of (0.98) when compared to other existing techniques. Though the new protocol shows better performance with reduced space complexity and latency and fixes flaws in 5G-AKA, its reliance on certain cryptographic approaches presents dangers going forward. To guarantee the resilience and dependability of the protocol in practical implementations, extensive tests in a variety of network circumstances are necessary. Although the protocol offers improved security measures, its dependence on particular cryptographic techniques might leave it vulnerable to future compromises of these techniques. Errors or vulnerabilities in the implementation that might be exploited are also a possibility. To make sure it doesn’t create unanticipated security issues or performance bottlenecks, the protocol’s performance under various network circumstances and large-scale deployments has to be carefully assessed. To verify its robustness and dependability in real-world applications, these evaluations are crucial. Future 5G studies ought to focus on managing mobility and sustainable practices, such as accurate UE motion forecasts to cut down on repetition.

## Data Availability

All data generated or analyzed during this study are included in this published article [and its supplementary information files].
